# Interleukin-6 in complications of prematurity: from biomarker to targeted therapy

**DOI:** 10.3389/fped.2026.1818320

**Published:** 2026-08-10

**Authors:** Shuzhe Xiao, Qi Zheng, Lingling Wang, Zhiqiu Wang, Guangliang Bi, Jie Yang

**Affiliations:** 1 Department of Neonatology, Nanfang Hospital, Southern Medical University, Guangzhou, China; 2 Guangzhou Women and Children's Medical Center, Guangzhou Medical University, Guangzhou, China; 3 The First Clinical Medical College of Southern Medical University, Guangzhou, China

**Keywords:** IL-6, prematurity complications, inflammation, immune cell, biomarker

## Abstract

Prematurity is a major cause of neonatal morbidity and mortality. Inflammatory imbalance is critical in preterm complications, and interleukin-6 (IL-6) plays a pivotal role in this dysregulation. Currently, there is no comprehensive review focusing specifically on IL-6 in the context of neonatal complications of prematurity. Thus, we review the involvement of IL-6 in various prematurity complications, exploring its mechanisms and clinical implications. We also discuss current and emerging therapies targeting IL-6, its receptor, and the trans-signaling pathway, focusing on the potential of these cytokines as therapeutic targets for the development of safe and effective treatments for neonatal complications of prematurity. However, while IL-6 shows consistent value as an early predictive biomarker, its translation into targeted therapy for preterm infants remains preliminary, with substantial gaps in safety and pharmacokinetic data that require further investigation.

## Highlights

- IL-6 serves as a predictive biomarker for sepsis, BPD, NEC, AKI, ROP, and HIE in preterm infants.
- Targeting IL-6, especially selective inhibition of trans-signaling, offers therapeutic promise.
- Safety data from pediatric and pregnancy cohorts support the translational potential of IL-6-targeted therapies in complications of prematurity.

## 1 Introduction

**Figure 1:**
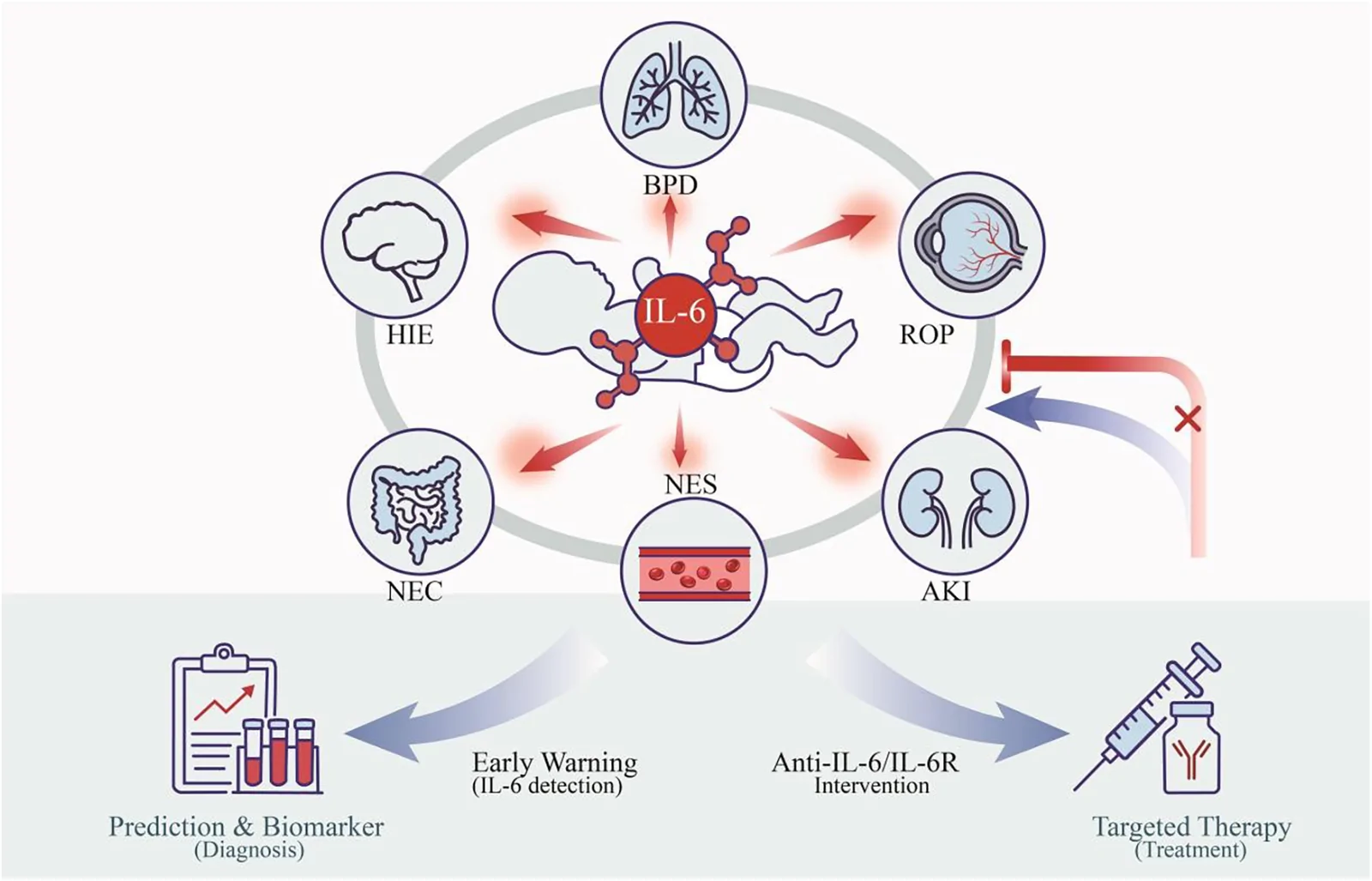
IL-6 and complications.

Worldwide, there are 13.4 million prematurity cases each year. Early birth complications greatly affect the health status of preterm babies and bring great mental and economic burdens to their families. In 2019, neonatal disorders were the leading cause of death among children under five years of age worldwide (1). Approximately 900,000 children died from complications of preterm birth, and many of the surviving preterm infants suffered from subsequent complications. Medical interventions, the maternal environmental factors, infection, and other factors are associated with preterm birth. Normal pregnancy involves dynamic changes in inflammatory responses and immune tolerance. Once inflammatory immune response and immune tolerance are imbalanced, the risk of preterm labor is greatly increased (2, 3). Interleukin–6 (IL–6) is associated with the onset of preterm labor, the progression of normal delivery, and the development of complications related to preterm birth (4, 5). Increased levels of IL–6 have been shown in bronchopulmonary dysplasia (BPD), neonatal septicemia (NES), retinopathy of prematurity (ROP), acute kidney injury (AKI), necrotizing enterocolitis (NEC), and hypoxic-ischemic encephalopathy (HIE). This evidence suggests that IL-6 is closely associated with the development of complications in preterm infants and has the potential to be an ideal predictor. The involvement of IL-6 in these complications is shown in Figure 1.

## 2 IL-6

Interleukin-6 (IL-6), a pivotal pro-inflammatory cytokine encoded on chromosome 7p15.3, transduces signals through a type I receptor complex composed of IL-6 receptor (IL-6R) (in membrane-bound or soluble form) and gp130. It exhibits pleiotropic functions, including the activation of B and T cells, induction of acute-phase protein production, and regulation of inflammation and cell differentiation (6). Crucially, IL-6 exerts distinct effects via two signaling pathways: the classical signaling through membrane-bound IL-6R is primarily associated with regenerative and anti-inflammatory activities, whereas trans-signaling via soluble IL-6R predominantly drives pro-inflammatory responses (7, 8). Compared to the limitations of other biomarkers, such as the IL-1 family (unreliable detection and unclear mechanisms), IL-8 (lack of specificity), C-reactive protein (CRP, delayed response and insufficient specificity), procalcitonin (PCT, low sensitivity), and TNF-α (complex detection requirements and non-specificity). IL-6 demonstrates superior potential due to its central role in neonatal inflammation, the early warning value offered by its dynamic changes, and the clear target ability of its signaling pathways. Therefore, this study will focus on elucidating the precise mechanisms of IL-6 in specific neonatal diseases and systematically evaluating its translational value from early diagnosis to targeted therapy (9–12). The dual role of IL-6 in health and disease is now understood to arise from its ability to signal via two distinct pathways, which often exert opposing effects: a protective classical pathway and a pathogenic trans-signaling pathway (13). The membrane-bound IL-6 receptor (mIL-6R) mediates classical signaling and is expressed only on a subset of cells, including hepatocytes, megakaryocytes, and some leukocytes. This route is principally important for IL-6's regenerative, anti-inflammatory, and host-defense actions, which include acute-phase protein synthesis and protection against bacterial infection. Trans-signaling, on the other hand, is mediated by the soluble IL-6 receptor (sIL-6R), which forms a complex with IL-6 and can activate almost any cell expressing gp130, a signal-transducing subunit that is ubiquitously expressed. This route is regarded as the major driver of chronic inflammation, autoimmunity, fibrosis, and tissue destruction (14).

## 3 Interleukin-6 family

**Figure 2:**
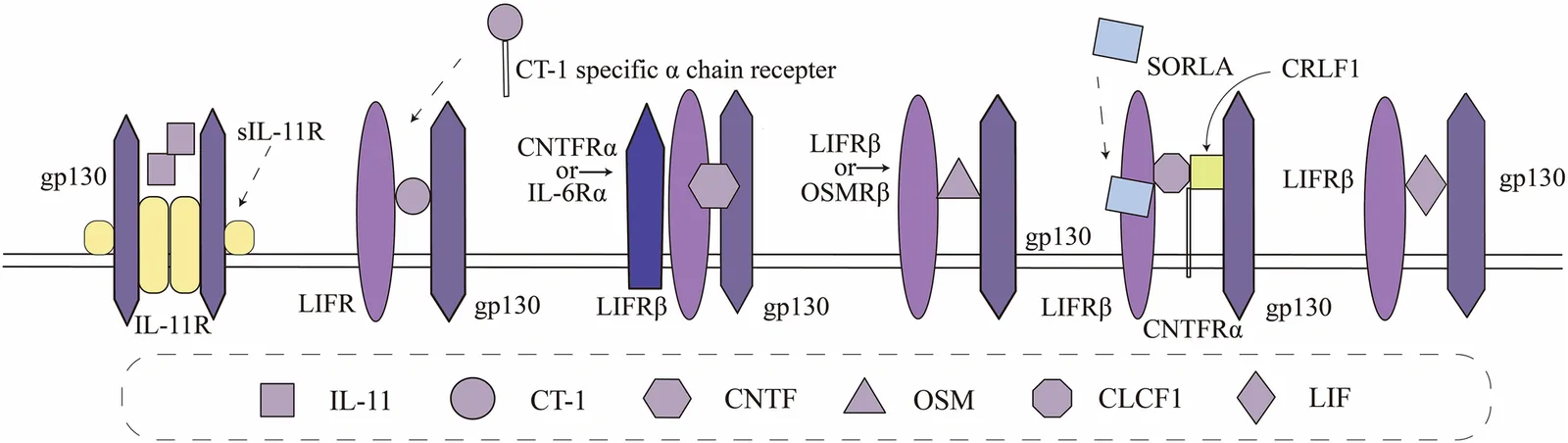
IL-6 family and their complexes. IL-11, interleukin-11; IL-11R, interleukin-11 receptor; sIL-11R, soluble IL-11 receptor; CT-1, cardiotrophin-1; CNTF, ciliary neurotrophic factor; CNTFRα, ciliary neurotrophic factor receptor α; OSM, oncostatin M; OSMRβ, oncostatin M receptor β; LIF, leukemia inhibitory factor; LIFRβ, leukemia inhibitory factor receptor β; CLCF1, cardiotrophin-like cytokine factor 1; CRLF1, cytokine receptor-like factor 1; gp130, glycoprotein 130; IL-6Rα, interleukin-6 receptor α.

The IL-6 cytokine family includes IL-6 and several related cytokines, such as IL-11, IL-27, IL-31, OSM (oncostatin M), CNTF (ciliary neurotrophic factor), CT-1 (cardiotrophin-1), LIF (leukemia inhibitory factor), and CLCF1 (cardiotrophin-like cytokine factor 1). Six members are shown in Figure 2. They are associated with haematopoiesis, neurological conditions, cancer, heart disease, and inflammatory diseases, among other conditions (15–24).

## 4 IL-6 and immune cells

IL-6 critically regulates macrophages and T cells. In macrophages, IL-6 suppresses pro-fibrotic differentiation in the liver (24), sustains a tumor-promoting feedback loop in lung adenocarcinoma (23), and drives M1-like polarization under hyperoxia, aggravating lung injury in BPD (25, 26). In T cells, IL-6 activates JAK2/STAT3 to affect the Th17/Treg balance (27, 28). IL-6 regulates Treg cells (29). IL-6 also drives B cells into long-lived plasma cells and naive T cells into Tfh cells (29, 30). In chronic intestinal inflammation, the IL-6/sIL-6R system modulates T cell resistance against apoptosis, and IL-6 produced by enteric neurons modulates iTreg formation and their RORγ proportion (31, 32).

## 5 Classical signaling pathways of IL-6

**Figure 3:**
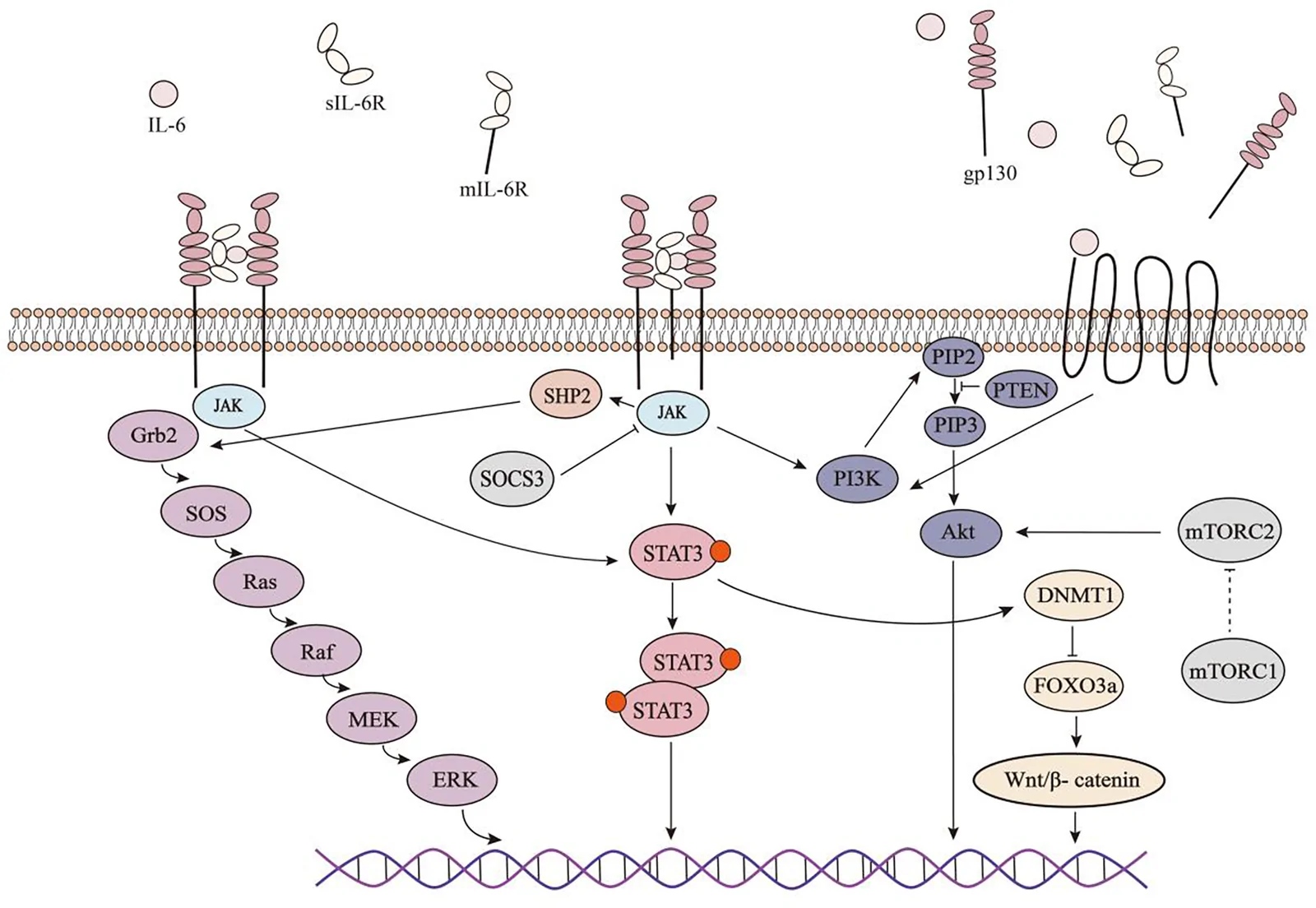
IL-6 signaling pathway. From left to right: 1. Ras/Raf/MEK/ERK pathway; 2. JAK/STAT signalling pathway; 3. PI3K/Akt pathway; 4. Wnt/β-catenin pathway. Suppressor of cytokine signaling-3 (SOCS3) inhibits JAK, mTORC1 (mTOR complex 1) indirectly inhibits mTORC2, PTEN inhibits the phosphorylation of phosphatidylinositol (4,5)-bisphosphate (PIP2) into phosphatidylinositol (3,4,5)-trisphosphate (PIP3). The orange dots represent phosphorylation events at key residues.

IL-6 plays a central role in the pathogenesis of neonatal diseases through its complex signaling network (Figure 3). Among this network, the JAK/STAT pathway serves as a core mechanism mediating its pro-inflammatory and pro-fibrotic effects. In pulmonary fibrosis, the activated JAK/STAT pathway not only directly promotes the release of inflammatory factors but is also a key driver of fibroblast to mesenchymal transition. This process leads to the transformation of pulmonary fibroblasts into myofibroblasts, exacerbating pulmonary fibrosis and impairing alveolar development (33). In NEC, YWHAB overexpression partially reversed the cellular impairment and inflammatory changes induced by miR-375–3p knockdown (34).

A different signaling route associated with IL-6 involves the PI3K/Akt pathway. The activation state of PI3K influences cell survival, cell cycle progression, differentiation, senescence, metabolism, and the critical events that regulate inflammatory responses to injury and infection (7). IL-6 and the PI3K/Akt pathway have been implicated in pulmonary fibrosis (35). In sepsis, sepsis-related bacteria induce monocyte-derived cytokine responses, including IL-6 (36). Separately, IFN-γ upregulates Δ42PD1 expression on monocytes through the PI3K/AKT pathway (37).

Furthermore, the Wnt/β-catenin pathway, crucial for development, engages in significant crosstalk with IL-6 signaling (38). In BPD, factors such as neutrophil extracellular traps (NETs) create a detrimental microenvironment characterized by the coexistence of inhibited Wnt/β-catenin signaling causing inflammation and fibrosis (39). This signaling imbalance represents a critical pathological basis for disrupted lung development and aberrant repair in preterm infants. Similarly, in ROP, hypoxia not only induces the production of classic inflammatory factors like IL-6, but genetic variants in the Wnt signaling pathway, commonly found in severe ROP and critical for retinal vascular development and repair, may also interfere with these processes, collectively contributing to neurovascular injury (40, 41).

## 6 IL-6 and complications of prematurity

### 6.1 Neonatal septicemia (NES)

NES is a systemic inflammatory disease. The process of inflammation is characterized not only by an increase in neutrophils, macrophages, and T-cells, but also by the release of pro-inflammatory mediators such as cytokines and chemokines. Due to the complexity of the inflammatory response in NES, the search for effective markers from a large number of inflammatory cells and inflammatory mediators is important to the treatment of NES (42). Serum levels of C-reactive protein (CRP) and IL-6 were found to be elevated in the majority of patients, and IL-6 is important for pro- and anti-inflammatory effects.

Current research focuses on biomarkers like IL-6, TNF-α, procalcitonin (PCT), amyloid A (SAA), and CRP for early NES diagnosis. Six hours after the onset of inflammation, IL-6 peaks and then decreases over the following 48 h, playing a role in the production of PCT and SAA. A study of 128 neonates with prenatal risk factors of infection (including 67 term and 61 preterm infants) found that cord blood IL-6 was more effective than CRP in predicting early-onset neonatal sepsis, with an AUC of 0.88 (43). Studies in preterm infants have identified multiple IL-6 thresholds for sepsis diagnosis: a saliva IL-6 level > 367.25 pg/mL combined with blood glucose > 67 mg/dL in one cohort (44); and in another cohort of 163 NICU patients (mean gestational age 28.7 ± 4.7 weeks), a serum IL-6 level < 130 pg/mL alone demonstrated higher sensitivity (100%) and positive predictive value (52%) for ruling out sepsis compared with combined biomarkers (45). IL-6 is widely considered to be an important biomarker in the development and the prediction of NES, but the threshold to determine the disease using IL-6 is still unknown.

IL-6 rises rapidly and reliably in early infection, making it a highly sensitive early warning marker for neonatal sepsis. However, reported cut-off values vary widely across studies due to differences in sample types and assay methods, and IL-6 elevation lacks specificity, as it also increases in other inflammatory conditions. These limitations do not diminish its value but highlight the need for standardized protocols and multi-biomarker approaches. Combining IL-6 with clinical parameters or disease-specific markers in large preterm cohorts will enhance its diagnostic accuracy.

### 6.2 Bronchopulmonary dysplasia (BPD)

BPD causes respiratory and neurological developmental deficits and contributes to increased healthcare costs for preterm infants. Several inflammatory factors and chemokines such as IL-6 are present in the alveolar lumen of preterm infants undergoing mechanical ventilation. IL-6 is associated with lung injury (46, 47). Another study shows that there is an increase in IL-6 in the plasma of children with BPD (48).

The increased plasma levels of IL-6 in BPD patients may be attributed to overexpression of IL-6 mRNA and suppressor of cytokine signaling-3 (SOCS3) deficiency, which leads to enhanced JAK/STAT activation, promotes M1 macrophage polarization, and mediates the inflammatory response (49). In hyperoxia, the gene encoding IL-6 was up-regulated nearly 20 times, which induced apoptosis of alveolar epithelial cells and aggravated lung injury in neonatal mice. The hyperoxic environment induced the expression of M1-like macrophages while inhibiting M2-like macrophages. It promotes alveolar epithelial type II cell (AT2) apoptosis and fibrosis, and affected AT2 homeostasis. These changes are closely related to the pathogenesis of BPD. In addition, Hirani et al. found that the development of alveoli was less affected in the IL-6 knockout mice than in wild-type mice. IL-6 knockout mice had fewer disorganized elastic fibers in the alveoli, more surface-active proteins, and AT2, suggesting a close relationship between IL-6 expression and lung dysplasia (25).

In clinical trials, Gao et al. found that cord blood IL-6 showed potential as a biomarker and therapeutic target for BPD (50), with an area under the receiver operating characteristic curve (AUROC) of 0.815 (95% CI: 0.753–0.877) in the differentiation of patients with moderate to severe BPD (Grade 2–3). IL-6 has the potential to become a biomarker and therapeutic target for BPD, but its predictive modeling accuracy and more specific relationship with BPD need to be further explored.

IL-6 levels are consistently elevated in infants who later develop BPD, supporting its role as an early predictor. A major unresolved issue is that most studies have measured total IL-6 without distinguishing between classical (protective) and trans-signaling (pathogenic) pathways, which may obscure the true pathological drivers. This mechanistic gap does not invalidate IL-6 as a biomarker but calls for pathway-specific assays to clarify which signaling arm should be targeted therapeutically. Future research should also establish optimal sampling timing relative to disease onset.

### 6.3 Necrotizing enterocolitis (NEC)

NEC is an avascular necrosis of the small intestine and colon that occurs in preterm infants. The pathogenesis of NEC mainly includes intestinal barrier immaturity, intestinal ischemia, changes in the intestinal flora, increased intestinal permeability, lowered intestinal immunity, genetic factors, and poor feeding. Currently, clinical diagnosis of NEC is mainly based on clinical symptoms, X-ray and ultrasound, but the diagnosis based on X-ray is not always accurate timely.

To investigate the role of IL-6, Egozi et al. used single-cell RNA sequencing to analyze the intestinal tissues of NEC. They found IL6, IL1B, and C-X-C motif chemokine ligand 8 (CXCL8) were increased (51). Yarciet al. designed an experiment on rats to inhibit IL-6 signaling and thus treat NEC (52). However, as Kim et al. noted, early and accurate diagnosis of NEC is challenging due to its unclear etiology and varied clinical presentation (53). The early prediction and diagnosis of NEC by inflammatory factors need further clinical evidence, and the diagnostic threshold needs to be thoroughly investigated.

IL-6 is consistently elevated in NEC and correlates with disease severity, making it a useful screening tool. However, its elevation overlaps with other inflammatory conditions such as sepsis, and reported cut-off values are inconsistent across studies. These challenges limit its use as a standalone diagnostic marker but do not preclude its clinical utility. Validating NEC-specific thresholds in prospective studies will improve diagnostic specificity.

### 6.4 Acute kidney injury (AKI)

Hyperoxia-induced renal injury in preterm infants, including proximal tubular injury, interstitial fibrosis, and glomerular injury, is associated with elevated IL-6 levels (54). Neonatal AKI is common and associated with adverse outcomes, and may predispose to long-term chronic kidney disease (55). Jasmine Mohr et al. knocked out the mice IL-6 gene and established a hyperoxia-induced kidney injury model, showing that chronic kidney diseases are dependent on the IL-6/STAT3 and Smad2 signaling pathways (56). Methyl-CpG binding protein 2 (Mecp2) plays a protective role in kidney tissue by inhibiting the IL-6/STAT3 axis, and it increased during AKI (57). However, the recovery of kidney function and tissue regeneration after kidney injury requires the involvement of the Wnt/β-catenin signaling pathway. Sustained activation of this pathway has been associated with chronic kidney disease (58). IL-6 may become a new target to protect preterm infants from kidney injury, and anti-IL-6 could be considered for the prevention and treatment of AKI in preterm infants.

Preclinical studies consistently demonstrate that IL-6 drives hyperoxia-induced kidney injury via the STAT3 pathway, establishing it as a biologically rational therapeutic target. The main translational challenge is that most evidence comes from animal models, and the dual role of IL-6 in kidney injury vs. repair has not been fully characterized in human preterm kidneys. These gaps do not undermine IL-6's potential; rather, they define a clear path forward: validating findings in human tissues and assessing long-term safety of IL-6 inhibition before clinical trials.

### 6.5 Retinopathy of prematurity (ROP)

ROP is a neurological disorder of the neonatal retina that can cause visual impairment and blindness. The development of ROP is associated with preterm birth and is influenced by variations in vascular endothelial growth factor (VEGF) expression, which are caused by factors such as fluctuations in exogenous oxygen levels, neonatal systemic inflammation, neonatal hyperglycemia, and neonatal sepsis (59). A study found that IL-6 levels in cord blood were positively correlated with the severity of ROP, where IL-6 >13.0 pg/ml could be used to support the diagnosis of ROP at stages 2-3/3-4 (41). TNF-a, IL-6, and IL-1 work together to stimulate the production of VEGF, which is important in the progression of ROP. Moreover, a study included the levels of IL-6 in cord blood and birth weight to construct a clinical prediction model (AUC = 0.840) that was able to predict ROP more accurately (60). Current clinical treatments for ROP include laser therapy and the use of anti-VEGF medicines, such as bevacizumab and ranibizumab. However, preterm infants treated with laser therapy have a higher risk of developing high myopia. On the other hand, anti-VEGF therapy carries a risk of adverse neurodevelopmental outcomes. Inhibiting IL-6 could become a new research area in the prevention and treatment of ROP.

Cord blood IL-6 levels correlate with ROP severity, and IL-6 is known to upregulate VEGF, a master driver of pathological angiogenesis, positioning IL-6 as an attractive upstream target for early intervention. Causal confirmation has not yet been achieved, and most published studies have small sample sizes with incomplete adjustment for confounders such as oxygen exposure and sepsis. These limitations are typical of an emerging field and do not invalidate the link; they simply highlight the need for well-powered prospective cohorts to establish causality and define the optimal treatment window.

### 6.6 Hypoxic ischemic encephalopathy (HIE)

HIE is a significant cause of neonatal mortality and neurodevelopmental impairments, affecting roughly 0.5–3 in 1,000 newborns (61). Early diagnosis of HIE lacks objective criteria, biomarkers such as glial fibrillary acidic protein, tau, neurofilament light chain, neuron-specific enolase, and S100 calcium-binding protein B in cerebrospinal fluid or blood of newborns have been suggested to be biomarkers (62). In a study of 180 neonates with moderate or severe HIE enrolled in the high-dose erythropoietin for asphyxia and encephalopathy trial, Juul et al. found that baseline values of C5a, IL-6, and NSE, as well as those collected on day 4 (IL-8, tau, and UCHL1), could predict mortality or neurodevelopmental impairments two years later, with an AUC of 0.79 (95% CI, 0.77–0.81, *P* = 0.01) (63). In addition, Toorell's team performed a clinical study examining the predictive potential of IL-6, which included 50 neonates with HIE, 50 neonates with asphyxia without HIE, and 50 healthy controls (64). They discovered that the levels of glial fibrillary acidic protein, tau, IL-6, and IL-8 in the cord blood from HIE infants were higher than in the control group, with all HIE cases showing higher IL-6 levels than the others (*P* = 0.008), correlating with disease severity. Therefore, IL-6 appears to have a predictive role for HIE.

IL-6 levels effectively distinguish HIE from uncomplicated asphyxia and correlate with neurological outcome severity, supporting its role as a valuable prognostic marker. A major evidence gap is that nearly all studies have been conducted in term infants, whereas preterm brain injury involves distinct inflammatory mechanisms. This gap does not imply that IL-6 is irrelevant in preterm HIE; instead, it strongly motivates preterm-focused research. Future studies should combine IL-6 with neuro-specific biomarkers in well-designed preterm cohorts to unlock its predictive potential. Table 1 summarizes the diagnostic performance of IL-6.

**Table 1:** Diagnostic performance of IL−6 as a biomarker for complications of prematurity.

Complication	Sample Source	Threshold (Cut-off)	Diagnostic Value (Sensitivity/Specificity/AUC)
Neonatal Sepsis (NES)	Cord Blood	-	AUC: 0.88
	Saliva	> 367.25 pg/mL	Sensitivity: High (in comb. with glucose)
Rule out sepsis	Serum	< 130 pg/mL	Sensitivity: 100%; PPV: 52%
Bronchopulmonary Dysplasia (BPD)	Cord Blood	-	AUC: 0.815 (95% CI: 0.753–0.877)
Retinopathy of Prematurity (ROP)	Cord Blood	> 13.0 pg/mL	ROP at stages 2–3/3–4(in comb. with TNF-α)
	Cord Blood	Birth weight and IL−6	AUC: 0.840
Hypoxic-Ischemic Encephalopathy (HIE)	Plasma	-	AUC: 0.79 (95% CI: 0.77–0.81)

For HIE, the study population consisted primarily of term infants; applicability to preterm infants requires further investigation.

## 7 IL-6-based therapeutic options

### 7.1 IL-6R antibody

Inhibiting IL-6R has been shown in many studies to be important in reducing inflammation in newborns. Ma Fei et al. discovered increased CCR9+ IL-17+ Treg in the peripheral blood of NEC patients and NEC mice, and that IL-6 plays a significant role in the differentiation of CCR9+ Treg cells to CCR9+ IL-17+ Treg cells. Increased circulating CCR9+ IL-17+ Treg cells in NEC mice may lead to elevated Th17 and IL-17+Treg cells in intestinal tissues (65). Compared to IgG antibody-treated NEC mice, NEC mice treated with anti-IL6R antibody showed a significant increase in Treg cells, a decrease in Th17 and IL-17+ Treg cells, an alleviation of the Treg/Th17 cell imbalance, a major reduction in STAT3 phosphorylation, and a significant increase in STAT5 phosphorylation. After treatment with IL6-R antibody, NEC mice had far higher survival rates (*p* < 0.01) and decreased severity scores (*p* *<* 0.05) (65).

Marcelo Farias-Jofre et al. discovered that anti-IL-6 receptor (aIL-6R) monoclonal antibody can be transmitted from mother to newborn through the maternal-foetal interface, preventing IL-1α-induced preterm labor and neonatal death (66). In mouse experiments, aIL-6R treatment prevented IL-1α-driven dysregulation of Il1a, Il1b, Tnf, and Lrg1 in the foetal brain, P2rx7 in foetal lungs, and Ccr5, Casp1, and Il12b in the small bowel, and Socs3, Ccr5, and Casp1 in the cecum, as well as Aim2 and Ccr5 in the colon, effectively prevented brain, lung and intestinal inflammation. aIL-6R regulates immune cells and gut flora in neonatal guts after IL-1α exposure (66).

With respect to inflammation-induced neurodevelopmental disorders, IL-6R antibody effectively inhibited IL-1a-induced reduction of neonatal biparietal diameter and significantly increased the passage rate of the negative geotaxis test, shortened the passage time and showed an improvement in neuromotor function (66).

### 7.2 IL-6 monoclonal antibody

Due to the lack of studies of siltuximab in prematurity complications, we have only described some of the therapeutic effects of siltuximab in children. Siltuximab is an anti-IL-6 monoclonal antibody. A 17-year-old boy who had both Idiopathic multicentric Castleman disease and connective tissue disorder was successfully treated by siltuximab (67). A 6-year-old girl with DOCK8 deficiency and severe herpetic infections achieved remission after treatment with siltuximab combined with glucocorticoids (68).

Olokizumab is also a monoclonal antibody against IL-6. The current researches on olokizumab are focused on rheumatoid arthritis. Olokizumab has shown promising therapeutic effects, but more comprehensive studies are needed (69, 70). Another IL-6 monoclonal antibody, WBP216, also showed promising results and safety in the treatment of rheumatoid arthritis (RA) (71).

### 7.3 Selective inhibition of IL-6 trans-signaling: a precision medicine approach

**Figure 4:**
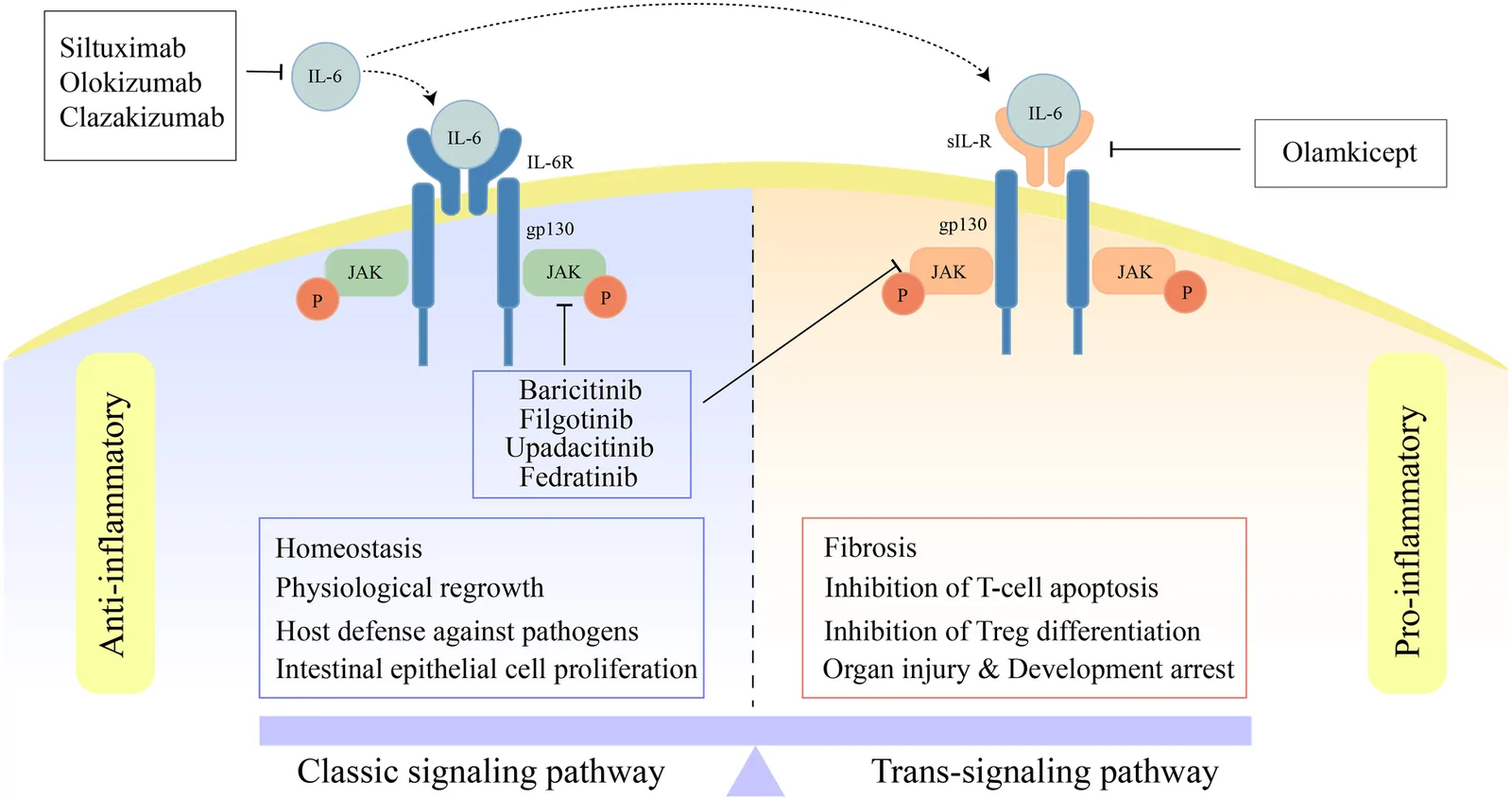
The classical signaling pathway and trans-signaling pathway of IL-6, as well as its inhibitors. Both classical signaling pathways and Trans-signaling pathways jointly maintain homeostasis. Inhibiting classical signaling pathways may lead to immune imbalance, while selectively inhibiting Trans-signaling pathways may address this issue. The orange circle indicates phosphorylation.

Sgp130Fc (Olamkicept) is a fusion protein that selectively inhibits IL-6 trans-signaling by binding to the IL-6/sIL-6R complex, thereby preventing its interaction with membrane-bound gp130 (72). This agent has shown good efficacy in patients with moderate to severe ulcerative colitis who do not respond to conventional treatment and in animal models of sepsis, pancreatitis, fracture healing and myocardial infarction (72). These findings highlight the translational potential of selective trans-signaling blockade, although its application in neonatal complications of prematurity remains unexplored. Figure 4 summarizes the two main signaling modes (classical and trans) and the key inhibitors (13, 73–75)

### 7.4 Targeting IL-6 for the treatment of childhood diseases in clinical studies

Tocilizumab, sarilumab, siltuximab, satralizumab, and other IL-6-targeting drugs have been used in a number of clinical trials for the treatment of children's diseases, including juvenile idiopathic arthritis, idiopathic multicentric Castleman disease and adamantinomatous, etc (Table 2).

**Table 2:** Targeting IL-6 for the treatment of childhood diseases in clinical studies.

ID	Study	Study Type	Age
Tocilizumab			
NCT02293837	Tocilizumab (TCZ) in New-onset Type 1 Diabetes	Interventional	6–17
NCT00868751	Single Patient Use of Tocilizumab in Systemic Onset Juvenile Idiopathic Arthritis	Interventional	2–16
NCT01104480	Tocilizumab for Relapsing Polychondritis	Interventional	12–15
NCT05233397	ACTEMRA® for the Treatment of Pediatric Adamantinomatous Craniopharyngioma	Interventional	1–25
NCT03970226	Tocilizumab in Children With ACP	Interventional	2–21
NCT02165345	Extension Study Evaluating the Safety and Efficacy of Subcutaneous Tocilizumab (RoActemra/​Actemra) Administration in Systemic and Polyarticular-Course Juvenile Idiopathic Arthritis	Interventional	2–18
NCT01904292	A Study of Subcutaneously Administered Tocilizumab in Participants With Systemic Juvenile Idiopathic Arthritis	Interventional	1–17
NCT01455701	A Study to Evaluate Pharmacokinetics and Safety of Tocilizumab (RoActemra/​Actemra) in Participants Less Than 2 Years Old With Active Systemic Juvenile Idiopathic Arthritis (sJIA)	Interventional	≤2
NCT02007239	Tocilizumab and Hemophagocytic Lymphohistiocytosis	Interventional	3mth–25
NCT01603355	Tocilizumab in the Management of Juvenile Idiopathic Arthritis Associated Uveitis	Interventional	2–17
NCT05164133	A Study Evaluating Tocilizumab in Pediatric Patients Hospitalized With COVID-19	Interventional	≤17
NCT01904279	A Study of Subcutaneously (SC) Administered Tocilizumab (TCZ) in Participants With Polyarticular-Course Juvenile Idiopathic Arthritis (pJIA)	Interventional	1–17
NCT00988221	A Study of Tocilizumab in Patients With Active Polyarticular Juvenile Idiopathic Arthritis	Interventional	2–17
NCT01667471	A Long-Term Extension Study of RoActemra/​Actemra (Tocilizumab) in Patients With Juvenile Idiopathic Arthritis Who Completed WA19977 Core Study	Interventional	9–18
Sarilumab			
NCT02776735	An Open-label, Ascending, Repeated Dose-finding Study of Sarilumab in Children and Adolescents With Polyarticular-course Juvenile Idiopathic Arthritis (pcJIA)	Interventional	2–17
NCT02991469	A Repeated Dose-finding Study of Sarilumab in Children and Adolescents With Systemic Juvenile Idiopathic Arthritis	Interventional	1–17
Siltuximab			
NCT04838860	Siltuximab In Siltuximab-RElapsed/ REfractory Multicentric Castleman Disease	Interventional	≥12
Satralizumab			
NCT04963270	A Study To Evaluate Efficacy, Safety, Pharmacokinetics, And Pharmacodynamics Of Satralizumab In Patients With Generalized Myasthenia Gravis	Interventional	≥12
NCT05271409	A Study to Evaluate the Efficacy, Safety, Pharmacokinetics, and Pharmacodynamics of Satralizumab in Patients With Myelin Oligodendrocyte Glycoprotein Antibody-Associated Disease	Interventional	≥12
NCT05503264	A Study To Evaluate The Efficacy, Safety, Pharmacokinetics, And Pharmacodynamics Of Satralizumab In Patients With Anti-N-Methyl-D-Aspartic Acid Receptor (NMDAR) Or Anti-Leucine-Rich Glioma-Inactivated 1 (LGI1) Encephalitis	Interventional	≥12
NCT02028884	Efficacy and Safety Study of Satralizumab (SA237) as Add-on Therapy to Treat Participants With Neuromyelitis Optica (NMO) and NMO Spectrum Disorder (NMOSD)	Interventional	12–74
Tocilizumab and Siltuximab			
≥NCT04850443	Treatment of IL-6 and Its Receptor Antagonists in Children's Severe Sepsis	Observational	29 Days to 18 Years

ACP, adamantinomatous craniopharyngioma; sJIA, systemic juvenile idiopathic arthritis; pJIA, polyarticular juvenile idiopathic arthritis; pcJIA, polyarticular-course juvenile idiopathic arthritis; NMO, neuromyelitis optica; NMOSD, neuromyelitis optica spectrum disorder.

## 8 Safety considerations of IL-6 targeted therapy in preterm infants

While IL-6 targeted medicines demonstrate potential efficacy, their safety profile in the vulnerable preterm newborn population warrants careful consideration (Table 3). The potential for transplacental transfer of monoclonal antibodies when provided during pregnancy is a major worry, as it may alter newborn immune function. Although preclinical studies indicate that anti-IL-6R antibodies can cross the placenta and may provide benefits such as preventing inflammation-induced preterm birth (66), clinical data in humans are limited, and theoretical risks such as neonatal cytopenias and potential blunting of the acute-phase response to infection persist (76). A recent retrospective cohort study gives important information about second and third-trimester exposure. All 25 pregnant women with severe COVID-19 treated with tocilizumab or sarilumab gave birth to live babies, but there was a high rate of premature delivery (16 of 26 neonates). Crucially, transitory newborn cytopenia was seen in 6 of the 19 neonates who underwent hematological testing. Although it was difficult to discern between therapeutic effects and preterm issues, anti-IL-6 transplacental transit could not be ruled out (77). Extrapolating from long-term safety data in pediatric populations, subcutaneous tocilizumab treatment in children with juvenile idiopathic arthritis for up to 5 years was associated with manageable adverse events, with infections being the most common and neutropenia occurring in a subset of patients without a clear link to subsequent serious infections (78). However, due to the immature immune system, distinct pharmacokinetics, and increased vulnerability of preterm infants, any future use of these biologics must be guided by rigorous pharmacokinetic/pharmacodynamic studies and vigilant monitoring for hematological, hepatic, and infectious complication.

**Table 3:** Summary of adverse effects associated with IL-6 pathway inhibitors.

Drug	Target	Adverse effects
Tocilizumab	IL-6 receptor	• Common: upper respiratory tract infections, nasopharyngitis, headache, elevated liver enzymes, injection site reactions. • Serious: neutropenia (grade 3–4), thrombocytopenia (rare), serious infections, hypersensitivity/anaphylaxis (rare). • Perinatal exposure: transient neonatal cytopenia (neutropenia, thrombocytopenia, lymphopenia) reported in 6/19 infants; causality difficult to distinguish from prematurity (71, 77, 78).
Sarilumab	IL-6 receptor	• Expected profile similar to tocilizumab: infections, neutropenia, transaminitis, injection site reactions. • Pregnancy exposure: one case reported; neonatal outcome uneventful, but data extremely limited (77).
Siltuximab	IL-6 (direct)	• Effective in idiopathic multicentric Castleman disease with connective tissue disorder and DOCK8 deficiency. • No significant adverse effects reported in these case reports (67, 68).
Olokizumab	IL-6 (direct)	• Based on adult RA trials: infections (pneumonia, cellulitis), neutropenia, elevated liver enzymes, injection site reactions. • No pediatric/perinatal safety data available (69, 70).
WBP216	IL-6 (direct)	• Early-phase adult data: generally well tolerated; mild adverse events including upper respiratory tract infection, injection site reactions. • Long-term and large-population safety data lacking (71).
Olamkicept (sgp130Fc)	IL-6/sIL-6R complex (selective trans-signaling inhibitor)	• Adult IBD trials: well tolerated; no observed increase in serious infections; no neutropenia signal; no anaphylaxis reported. • Theoretical advantage: preserves classical (protective) signaling; may carry lower infection risk than non-selective IL-6/IL-6R blockade. • No neonatal/perinatal data (72, 79).

## 9 Summary and future

This review describes the role of IL-6 in immune cells and the research results on IL-6 signaling pathways, as well as its role in common neonatal diseases such as NES, BPD, NEC, AKI, ROP, and HIE. At the end of the article, we listed the clinical trials targeting IL-6 for the treatment of childhood diseases and looked forward to the future. Although there is a lack of clinical studies specifically targeting IL-6 for the treatment of prematurity complications, mouse studies related to complications of prematurity and clinical trials targeting IL-6 for conditions such as juvenile idiopathic arthritis and lymphohistiocytosis demonstrate the potential of targeting IL-6 in treating preterm complications. Patients with IL-6-related diseases may be treated with IL-6-targeted drugs in the future.

As noted in each disease subsection, common limitations exist regarding cut-off standardization, disease specificity, pathway distinction, and population applicability. Beyond these limitations, a fundamental conceptual advance in the field is the recognition that IL-6 signals via two distinct pathways with opposing roles. The classical pathway mediates host defense and tissue repair, whereas the trans-signaling pathway drives pathological inflammation. This dichotomy has profound implications for future therapeutic development. Selective inhibition of trans-signaling using agents such as sgp130Fc (Olamkicept) represents a precision medicine approach that could theoretically preserve protective immunity while suppressing harmful inflammation, a particularly attractive strategy for preterm infants whose immune and organ systems are still developing. Preclinical studies in neonatal models of BPD, NEC, and perinatal brain injury are urgently needed to test this hypothesis.

Another critical knowledge gap is the complete absence of pharmacokinetic and pharmacodynamic data for IL-6 targeted biologics in preterm infants. Weight- and gestational age-appropriate dosing regimens cannot be rationally designed without such data. Furthermore, the long-term neurodevelopmental and immunological outcomes following perinatal exposure to IL-6 inhibitors remain unknown. Prospective registries and collaborative neonatal research networks will be essential to address these questions.

In summary, IL-6 demonstrates consistent value as a predictive biomarker for several complications of prematurity, but its translation into a therapeutic target remains uncertain due to the knowledge gaps discussed above. Future research should prioritize standardizing cut-off values, validating multi-biomarker panels in preterm cohorts, and conducting preclinical safety studies before considering any clinical application of IL-6-targeted therapies.
