# Correction: Interleukin−6 in complications of prematurity: from biomarker to targeted therapy

**DOI:** 10.3389/fped.2026.1965753

**Published:** 2026-09-10

**Authors:** Shuzhe Xiao, Qi Zheng, Lingling Wang, Zhiqiu Wang, Guangliang Bi, Jie Yang

**Affiliations:** 1 Department of Neonatology, Nanfang Hospital, Southern Medical University, Guangzhou, China; 2 Guangzhou Women and Children’s Medical Center, Guangzhou Medical University, Guangzhou, China; 3 The First Clinical Medical College of Southern Medical University, Guangzhou, China

**Keywords:** IL-6, prematurity complications, inflammation, immune cell, biomarker

Error in figure/table

There was a mistake in Table 1 as published. The data for Neonatal Sepsis (NES), Rule out sepsis, Retinopathy of Prematurity (ROP), and Hypoxic-Ischemic Encephalopathy (HIE) were incorrect. Specifically, the AUC for NES cord blood was given with a 95% CI; the “Rule out sepsis” row was missing; the ROP outcome was misstated as “Positive correlation with severity” and “Birth weigh” was misspelled; and the HIE plasma entry incorrectly read “Baseline levels” instead of a dash. The corrected Table 1 appears below.

**Table 1:** Diagnostic performance of IL−6 as a biomarker for complications of prematurity.

Complication	Sample Source	Threshold (Cut-off)	Diagnostic Value (Sensitivity/Specificity/AUC)
Neonatal Sepsis (NES)	Cord Blood	-	AUC: 0.88
	Saliva	> 367.25 pg/mL	Sensitivity: High (in comb. with glucose)
Rule out sepsis	Serum	< 130 pg/mL	Sensitivity: 100%; PPV: 52%
Bronchopulmonary Dysplasia (BPD)	Cord Blood	-	AUC: 0.815 (95% CI: 0.753–0.877)
Retinopathy of Prematurity (ROP)	Cord Blood	> 13.0 pg/mL	ROP at stages 2–3/3–4(in comb. with TNF-α)
	Cord Blood	Birth weight and IL−6	AUC: 0.840
Hypoxic-Ischemic Encephalopathy (HIE)	Plasma	-	AUC: 0.79 (95% CI: 0.77–0.81)

For HIE, the study population consisted primarily of term infants; applicability to preterm infants requires further investigation.

Text correction

The word “suggest” lacked an “s” and should be “suggests”. A correction has been made to the section Introduction, Paragraph 1:

“Worldwide, there are 13.4 million prematurity cases each year. Early birth complications greatly affect the health status of preterm babies and bring great mental and economic burdens to their families. In 2019, neonatal disorders were the leading cause of death among children under five years of age worldwide (1). Approximately 900,000 children died from complications of preterm birth, and many of the surviving preterm infants suffered from subsequent complications. Medical interventions, the maternal environmental factors, infection, and other factors are associated with preterm birth. Normal pregnancy involves dynamic changes in inflammatory responses and immune tolerance. Once inflammatory immune response and immune tolerance are imbalanced, the risk of preterm labor is greatly increased (2, 3). Interleukin−6 (IL−6) is associated with the onset of preterm labor, the progression of normal delivery, and the development of complications related to preterm birth (4, 5). Increased levels of IL−6 have been shown in bronchopulmonary dysplasia (BPD), neonatal septicemia (NES), retinopathy of prematurity (ROP), acute kidney injury (AKI), necrotizing enterocolitis (NEC), and hypoxic-ischemic encephalopathy (HIE). This evidence suggests that IL-6 is closely associated with the development of complications in preterm infants and has the potential to be an ideal predictor. The involvement of IL-6 in these complications is shown in Figure 1.”

The first paragraph of section 5. Classical signaling pathways of IL-6 contained a redundant sentence that was incorrectly included and has been removed. A correction has been made to the section 5. Classical signaling pathways of IL-6, Paragraph 1:

“IL-6 plays a central role in the pathogenesis of neonatal diseases through its complex signaling network (Figure 3). Among this network, the JAK/STAT pathway serves as a core mechanism mediating its pro-inflammatory and pro-fibrotic effects. In pulmonary fibrosis, the activated JAK/STAT pathway not only directly promotes the release of inflammatory factors but is also a key driver of fibroblast to mesenchymal transition. This process leads to the transformation of pulmonary fibroblasts into myofibroblasts, exacerbating pulmonary fibrosis and impairing alveolar development (33). In NEC, YWHAB overexpression partially reversed the cellular impairment and inflammatory changes induced by miR-375–3p knockdown (34).”

In the second paragraph of this subsection, the word “In” was incorrectly capitalised and should be “in”, and the phrase “the most important biomarker” has been revised to “an important biomarker”. A correction has been made to the section 6.1 Neonatal septicemia (NES), Paragraph 2:

“Current research focuses on biomarkers like IL-6, TNF-α, procalcitonin (PCT), amyloid A (SAA), and CRP for early NES diagnosis. Six hours after the onset of inflammation, IL-6 peaks and then decreases over the following 48 h, playing a role in the production of PCT and SAA. A study of 128 neonates with prenatal risk factors of infection (including 67 term and 61 preterm infants) found that cord blood IL-6 was more effective than CRP in predicting early-onset neonatal sepsis, with an AUC of 0.88 (43). Studies in preterm infants have identified multiple IL-6 thresholds for sepsis diagnosis: a saliva IL-6 level > 367.25 pg/mL combined with blood glucose > 67 mg/dL in one cohort (44); and in another cohort of 163 NICU patients (mean gestational age 28.7 ± 4.7 weeks), a serum IL-6 level < 130 pg/mL alone demonstrated higher sensitivity (100%) and positive predictive value (52%) for ruling out sepsis compared with combined biomarkers (45). IL-6 is widely considered to be an important biomarker in the development and the prediction of NES, but the threshold to determine the disease using IL-6 is still unknown.”

The original version of this article has been updated.

